# Crizotinib Efficacy After Progression With Entrectinib in ROS1-Positive Lung Cancer: A Case Report

**DOI:** 10.7759/cureus.27828

**Published:** 2022-08-09

**Authors:** Hakan Taban, Deniz Can Guven, Saadettin Kılıçkap

**Affiliations:** 1 Department of Medical Oncology, Hacettepe University Cancer Institute, Ankara, TUR; 2 Department of Medical Oncology, Istinye University Faculty of Medicine, Istanbul, TUR

**Keywords:** targeted therapy, lung cancer, ros1, entrectinib, crizotinib

## Abstract

Crizotinib and entrectinib are approved tyrosine kinase inhibitors by the FDA to treat advanced-stage ROS1-positive non-small cell lung cancer (NSCLC). Although, entrectinib could be used after crizotinib, it is unknown whether crizotinib is effective after entrectinib. We report a case of NSCLC with ROS1 rearrangement that achieved a nearly complete response with crizotinib in the second-line treatment after progression with entrectinib. A 22-year-old Caucasian non-smoker female patient was diagnosed with stage IV non-squamous lung cancer with ROS1 positivity. We started on entrectinib as first-line therapy. Due to progression in the 10th month of treatment, entrectinib was stopped and crizotinib was started as a second-line treatment. At the end of the third month of the treatment, a nearly complete response was obtained in the follow-up imaging. The patient is still being followed up with crizotinib and is in the 15th month of treatment. Based on our experience, crizotinib can be an option as second-line therapy in patients who are treated with entrectinib in the first line, especially in patients without brain metastasis.

## Introduction

ROS1 gene rearrangements/fusions occur in 1-2% of patients with non-small-cell lung cancer (NSCLC) [1]. It is frequently seen in young females, never/light smokers, and patients with Asian ethnicities [2]. Crizotinib and entrectinib are approved tyrosine kinase inhibitors (TKIs) to treat advanced-stage ROS1-positive NSCLC [1,2]. The median progression-free survival (PFS) is around 19 months with both approved TKIs in first line [3,4]. However, while entrectinib could be used after crizotinib, it is unknown whether crizotinib is effective after entrectinib due to the lack of clinical trials and case reports/series. Here, we report a case of NSCLC with ROS1 rearrangement that achieved a nearly complete response with crizotinib in the second-line treatment after progression with entrectinib.

## Case presentation

**Figure 1:**
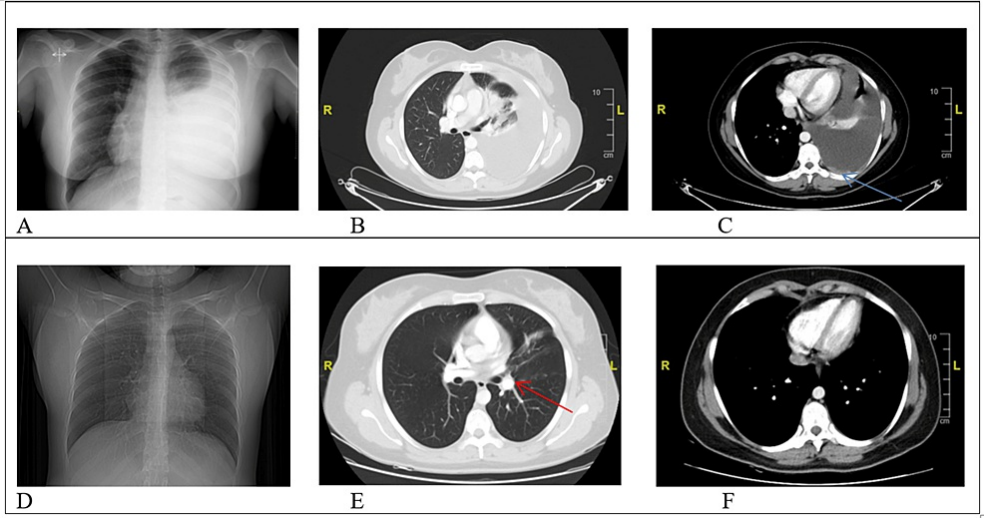
Chest X-ray and CT sections before crizotinib treatment (A, B, C) and after crizotinib treatment (D, E, F) (A) left-sided massive pleural effusion and right-sided mediastinal shift on chest X-ray; (B) and (C): left-sided massive pleural effusion and pleural metastasis (blue arrow) are remarkable on CT sections; (D): left-sided massive pleural effusion completely disappeared on chest X-ray; (E) and (F): massive pleural effusion and pleural metastasis disappeared on CT sections. Also, a primary tumor with a diameter of 2 cm in the left lung (red arrow) became detectable.

A 22-year-old Caucasian non-smoker female patient presented with complaints of dyspnea and cough in March 2020. Radiological imaging revealed left-sided massive pleural effusion, multiple pleural metastases, and a lesion compatible with primary lung cancer. There was no brain metastasis in brain imaging. Thoracentesis for cytologic evaluation and symptom relief and a biopsy from the primary lesion was performed. Then, the patient was diagnosed with cT3 cNx cM1a, stage IV non-squamous lung cancer according to American Joint Committee on Cancer (AJCC) 8th edition. In molecular tests, we detected ROS1 rearrangement with the fluorescence in-situ hybridization test, and the fusion partner was CD74. We started on entrectinib 600 mg/day as first-line therapy and obtained a partial response at the first radiological control. Although the patient's treatment compliance and tolerance were well, treatment-related side effects of grade 2 dyspepsia and weight gain were observed. In the ninth month of treatment, an increase in left-sided pleural effusion and pleural thickening were revealed. The patient was asymptomatic and clinically stable, so we continued entrectinib treatment for one more month and planned a thorax computed tomography (CT) scan. After one month, the patient had dyspnea and radiologic progression in the new thorax CT scan. Malignant cells were detected in the cytological evaluation of the pleural effusion. We could not perform any entrectinib-resistance mechanism study. We changed entrectinib with crizotinib 250 mg twice daily as second-line therapy. Before the crizotinib treatment, there was no brain metastasis in cranial magnetic resonance imaging (MRI). Thoracentesis was performed twice in the first month of crizotinib treatment due to dyspnea secondary to pleural effusion. At the end of the first month, the patient's dyspnea regressed, and pleural effusion decreased on chest X-ray. The crizotinib was well tolerated with no adverse events other than grade 1 alanine aminotransferase, aspartate aminotransferase elevation, and grade 1 nausea/vomiting. Thoraco-abdominopelvic CT scans and brain MRI were performed at the end of the third month of treatment. The control imaging demonstrated regression of the primary tumor, near-complete regression of pleural metastases, and complete resolution of the pleural effusion (Figure 1).

Two millimetric brain metastases were observed in the 12th month of crizotinib treatment. Stereotactic radiosurgery was performed for brain metastases. The patient is in the 15th month of treatment and is still being followed up with crizotinib.

## Discussion

We reported a response to crizotinib as a second-line treatment after progression with entrectinib in a ROS1-positive lung cancer patient. While the recommended treatment options after progression with entrectinib are lorlatinib and systemic chemotherapy ± immunotherapy in the guidelines [1,2], we opted out to try another TKI approved in the first line due to patient preference and not having access to lorlatinib. To the best of our knowledge, this is the first report of crizotinib efficacy after entrectinib.

Crizotinib is an oral TKI that inhibits ROS/ALK and MET. It is the first agent to be approved to treat advanced-stage NSCLC patients with ROS1 rearrangement based on the results of the phase I PROFILE 1001 study in 2016 [3]. This study included 50 treatment-naive and chemotherapy-treated patients with advanced-stage NSCLC with ROS1 positivity. The disease control rate (DCR) was 90%, median PFS was 19.2 months, and the objective response rate (ORR) was 72% [3]. In the updated results in 2019, the median overall survival was reported to be 51.4 months [5]. The PROFILE 1001 and following European Trial on Crizotinib in ROS1 Translocated Lung Cancer (EUCROSS) [6] studies with crizotinib enrolled TKI-naïve patients only, creating a knowledge gap in situations we encountered in our patient.

Entrectinib is a relatively new TKI that inhibits ROS1, NTRK, and ALK and has a good blood-brain barrier penetration in contrast to crizotinib [7]. Integrated analysis of three ongoing phase-1/2 trials of entrectinib in 53 patients with TKI-naive ROS1-positive NSCLC showed 19 months median PFS and 77.4% ORR [4]. Although acquired crizotinib resistance is better understood, our knowledge of resistance to entrectinib is very limited [8]. In a preclinical study, researchers have demonstrated the acquisition of KRAS G12C, sustained ERK activation, and the amplification of KRAS and FGF3 as the entrectinib resistance mechanisms [7]. Interestingly, they found no secondary mutations in the ROS1 kinase domain. Liquid biopsy was performed at progression in ROS1-positive lung cancer patients in the STARTRK-2 study to understand the genomic landscape of entrectinib resistance and acquired ROS1 resistance mutations (G2032R and F2004C/I) were detected in five (28%) patients [9]. We don't know the exact mechanism of our patient's good response to crizotinib after entrectinib. This may be related to the difference in binding domains of the drugs or the development of a crizotinib-sensitive mutation.

At the time of progression, tissue or liquid biopsy could be performed to evaluate for resistance mutations or bypass signaling pathways. However, we did not perform tissue or liquid biopsy after progression with entrectinib. We started crizotinib treatment in our patient due to a lack of access to lorlatinib and the patient's unwillingness to undergo chemotherapy. We consider this situation as a limitation in the management of the patient. The prioritization of the re-biopsies during progression is vital to choosing the appropriate drug and delineating the resistance mechanisms to next-generation TKIs.

## Conclusions

Treatment options have been increasing in lung cancer patients with ROS1 rearrangement. In the first line, crizotinib and entrectinib (in the accessible countries) are the recommended treatments, while lorlatinib or chemotherapy ± immunotherapy are the second-line options. Based on experience in our case, crizotinib can be an option as second-line therapy in patients who receive entrectinib in the first line, especially in patients without brain metastasis. Further research is needed to elucidate specific ROS1 mutations and mechanisms of resistance to TKIs for optimization of treatment sequencing in NSCLC with ROS1 rearrangement.
